# Protocol for a randomised controlled unblinded feasibility trial of HD-DRUM: a rhythmic movement training application for cognitive and motor symptoms in people with Huntington’s disease

**DOI:** 10.1136/bmjopen-2023-082161

**Published:** 2024-07-31

**Authors:** Vasileios Ioakeimidis, Monica Busse, Cheney J G Drew, Philip Pallmann, Guy B Watson, Derek Jones, Marco Palombo, Robin Schubert, Anne E Rosser, Claudia Metzler-Baddeley

**Affiliations:** 1 Cardiff University Brain Research Imaging Centre (CUBRIC), School of Psychology, Cardiff University, Cardiff, UK; 2 Centre for Trials Research, School of Medicine, Cardiff University, Cardiff, UK; 3 HD Voice, Huntington's Disease Association, Liverpool, UK; 4 School of Computer Science and Informatics, Cardiff University, Cardiff, UK; 5 George-Huntington-Institute, Muenster, Germany; 6 Cardiff Brain Repair Group, Cardiff, UK; 7 Department of Psychological Medicine and Clinical Neurosciences, School of Medicine, Cardiff University, Cardiff, UK

**Keywords:** Feasibility Studies, Randomized Controlled Trial, Magnetic Resonance Imaging

## Abstract

**Introduction:**

Huntington’s disease (HD) is an inherited neurodegenerative disease causing progressive cognitive and motor decline, largely due to basal ganglia (BG) atrophy. Rhythmic training offers promise as therapy to counteract BG-regulated deficits. We have developed HD-DRUM, a tablet-based app to enhance movement synchronisation skills and improve cognitive and motor abilities in people with HD. This paper outlines a randomised controlled unblinded trial protocol to determine the feasibility of a larger effectiveness trial for HD-DRUM. Additionally, the trial investigates cognitive and motor function measures, along with brain microstructure, aiming to advance our understanding of the neural mechanisms underlying training effects.

**Methods, design and analysis:**

50 individuals with HD, confirmed by genetic testing, and a Total Functional Capacity (TFC) score of 9–13, will be recruited into a two-arm randomised controlled feasibility trial. Consenting individuals with HD will be randomised to the intervention group, which entails 8 weeks of at-home usage of HD-DRUM or a usual-activity control group. All participants will undergo cognitive and motor assessments, alongside ultra-strong gradient (300 mT/m) brain microstructural MRI before and after the 8-week period. The feasibility assessment will encompass recruitment, retention, adherence and acceptability of HD-DRUM following prespecified criteria. The study will also evaluate variations in cognitive and motor performance and brain microstructure changes resulting from the intervention to determine effect size estimates for future sample size calculations.

**Ethics and dissemination:**

The study has received favourable ethical opinion from the Wales Research Ethics Committee 2 (REC reference: 22/WA/0147) and is sponsored by Cardiff University (SPON1895-22) (Research Integrity, Governance and Ethics Team, Research & Innovation Services, Cardiff University, second Floor, Lakeside Building, University Hospital of Wales, Cardiff, CF14 4XW). Findings will be disseminated to researchers and clinicians in peer-reviewed publications and conference presentations, and to participants, carers and the general public via newsletters and public engagement activities. Data will be shared with the research community via the Enroll-HD platform.

**Trial registration number:**

ISRCTN11906973.

**STRENGTHS AND LIMITATIONS OF THIS STUDY**

- HD-DRUM is a remotely accessible, tablet-based training tool that can be used at home and allows the objective assessment of adherence and training effects.
- The use of gamification to match users’ practice to a level appropriate to their abilities is expected to increase adherence and acceptability of HD-DRUM by avoiding frustration and boredom due to overchallenge and underchallenge.
- This trial will assess the feasibility of a future fully powered effectiveness randomised controlled trial and any modifications that may need to be implemented in HD-DRUM.
- The randomised controlled study design will allow the estimation of training-induced variability of changes in clinical and brain imaging measures, using state-of-the-art ultra-strong gradient 3T MRI.
- Due to the nature of the intervention and limited resources, researchers and participants are not blinded to group allocation.

## Introduction

### Background and rationale

 Huntington’s disease (HD) is a genetic neurodegenerative disease that leads to the progressive loss of cognitive and motor abilities, largely due to atrophy in the basal ganglia (BG)^1^ and other brain regions including the prefrontal cortex and frontostriatal connections.^2 3^ Striatal atrophy^1^ and microstructural alterations in white matter (WM) connections^2^ with associated cognitive control dysfunctions^3^,^5^ occur many years before the clinical onset of motor symptoms and will eventually negatively impact a person’s ability to live independently.^6^

Currently, there are no disease-modifying treatments for HD and symptomatic therapies are limited with significant side effects.^7 8^ There are no HD-specific cognitive interventions and only very few studies have investigated cognition-oriented interventions in people with HD.^9^

Rhythmic auditory stimulation (RAS) is a form of neurological music therapy^10^ that uses rhythmic beats as external cues to trigger movements.^11^ RAS has been found to improve gait and mobility in people with Parkinson’s disease (PD)^11^,^13^ and holds potential as an intervention for improving attention and executive functions.^12 14^ While PD and HD are clinically distinct diseases, they are both associated with BG neurodegeneration and deficits in interval timing and spontaneous rhythm generation.^15^ The BG is considered to play a key role in the prediction of upcoming events by internally generating temporal pacing signals.^16^,^18^ Together with the cerebellum and cortical areas involved in sensory, motor and attention processing, they form an extended network of brain regions that supports rhythmic sensory processing and movement generation.^16^ RAS interventions are proposed to work by compensating for the loss of BG-generated timing and rhythm signals with external rhythmic cueing.^11^ However, the actual neural mechanisms of rhythmic processing as well as the clinical effects of RAS interventions in people with HD remain unknown.

Previously, we explored 8 weeks of bongo drumming (~15 min five times per week) as a therapeutic tool for people with HD.^19 20^ We observed performance improvements in executive function tasks and changes in WM microstructural measurements of callosal and cortico-putamen connections.^19 20^ Although these initial pilot results were promising, they were preliminary in nature and necessitate validation through a fully powered randomised controlled trial (RCT). This study aims to investigate the feasibility of a larger RCT, as well as to explore any indicative effects and the neural mechanisms that may be responsible for potential clinical advantages associated with RAS.

For this purpose, we have codesigned with people with HD, a tablet-based RAS training application (app) (HD-DRUM)^21^ that is suitable for clinical evaluation because it allows the accurate quantification of training progression and adherence and uses gamification to match individual performance levels to training difficulty with the aim to increase acceptability. As HD-DRUM can be used at home it holds the potential of expanding intervention delivery to greater participant numbers for an effectiveness trial.

Here, we describe the protocol of an RCT to determine the feasibility of a larger effectiveness RCT into the clinical effects of HD-DRUM in people with HD. The trial will also explore training-induced changes in cognitive and motor functions as well as in MRI measures of morphology and microstructure of cortical and subcortical brain regions involved in rhythmic processing (BG, cerebellum, auditory and motor cortices). The clinical and neural effects of 8 weeks of HD-DRUM will be compared with a usual-activity control group in people with HD that will allow us to explore whether HD-DRUM can improve on current practice.

HD-DRUM has the potential to be a future training tool for slowing cognitive and motor decline in people with HD, alongside candidate disease-modifying treatments that are currently under consideration. Even a small delay in disease progression would translate into direct benefits for the quality of life of patients and their families.

### Primary objective

To assess the feasibility of a larger effectiveness RCT investigating 8 weeks of at-home HD-DRUM intervention compared with usual activities in people with HD.

### Secondary objectives

To gain estimates of variability and absolute change before and after 8 weeks of HD-DRUM compared with usual-activity control in the following measures:

1. Performance measures in cognitive and motor tasks for sample size calculations for a future RCT.
2. The means of grey and WM microstructure in BG and cerebellar brain networks^3^ to explore the neural mechanisms that underpin any training effects.
3. The means of performance measures in cognitive and motor tasks and of grey and WM microstructure in BG and cerebellar brain networks^3^ to inform about disease-specific neural mechanisms including compensatory processes.

### Trial design

**Figure 1:**
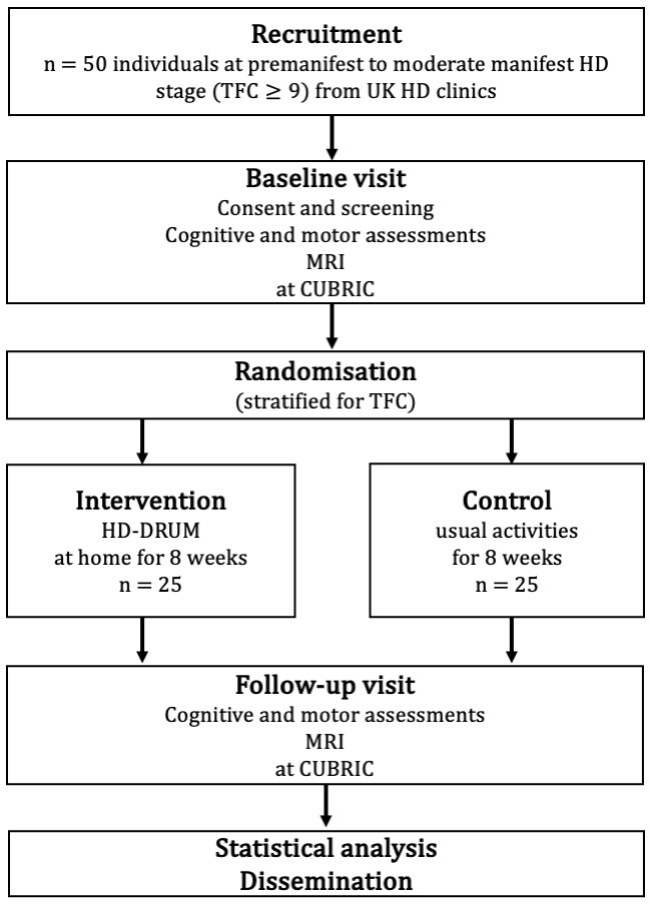
Schematic overview of the two-arm randomised controlled feasibility trial. CUBRIC, Cardiff University Brain Research Imaging Centre; HD, Huntington’s disease; TFC, Total Functional Capacity.

We will conduct a two-arm randomised controlled feasibility trial of individuals with HD randomised to 8 weeks of HD-DRUM intervention or usual-activity control (figure 1).

## Methods

### Patient and public involvement

This study was developed with members of HD Voice, the patient and public involvement (PPI) group of the UK HD Association (HDA). PPI members are involved in the management and oversight of the study as members of the study management and steering groups. The HD-DRUM intervention was codesigned with people with HD. PPI members will contribute to the interpretation and the dissemination of the study findings.

### Participants, interventions and outcomes

#### Study setting

Participants will be identified, approached and recruited via specialist HD clinics in the UK. All baseline and follow-up clinical and MRI assessments will take place at the Cardiff University Brain Research Imaging Centre (CUBRIC). Participants will engage individually with the HD-DRUM intervention at home.

#### Recruitment

Eligible individuals will be identified by their clinician during their annual visit. Individuals interested in the trial will receive the patient information sheet (PIS). They will be asked to provide consent for the researchers to contact them to find out if they wish to proceed with the study. If they decide to participate, during their first appointment at CUBRIC they will be asked to provide informed written consent and complete the study assessments. Participants will be reimbursed for their travel expenses and any overnight accommodation.

The study will also be advertised on posters in clinic waiting rooms and via newsletters and websites of Health and Care Research Wales (HCRW) and the HDA UK. In these instances, interested parties must first contact one of the participating HD centre teams for further information.

#### Eligibility criteria

Participants will be eligible to take part in the study if they meet all of the inclusion criteria and none of the exclusion criteria.

#### Inclusion criteria

Individuals over the age of 18 years with a good command of the English language who fulfil the following criteria:

1. Premanifest or manifest HD as confirmed by genetic testing for the presence of the mutant huntingtin allele.
2. A Unified Huntington’s Disease Rating Scale (UHDRS) TFC^22^ score between 9 and 13.
3. Who participate in the Enroll-HD^23^ observational study (former Registry) (REC no 04/WSE05/89) or in routine clinical assessment of their TFC score.

#### Exclusion criteria

A history of any other neurological condition and/or an inability to provide informed consent. MRI contraindications (eg, pacemakers, stents) will preclude participation in the MRI but not the training part.

#### Intervention

The HD-DRUM app is a computerised version of the drumming training intervention we have previously devised.^19 20^ A detailed description of HD-DRUM according to the Template for Intervention Description and Replication has been published.^21 24^

**Figure 2:**
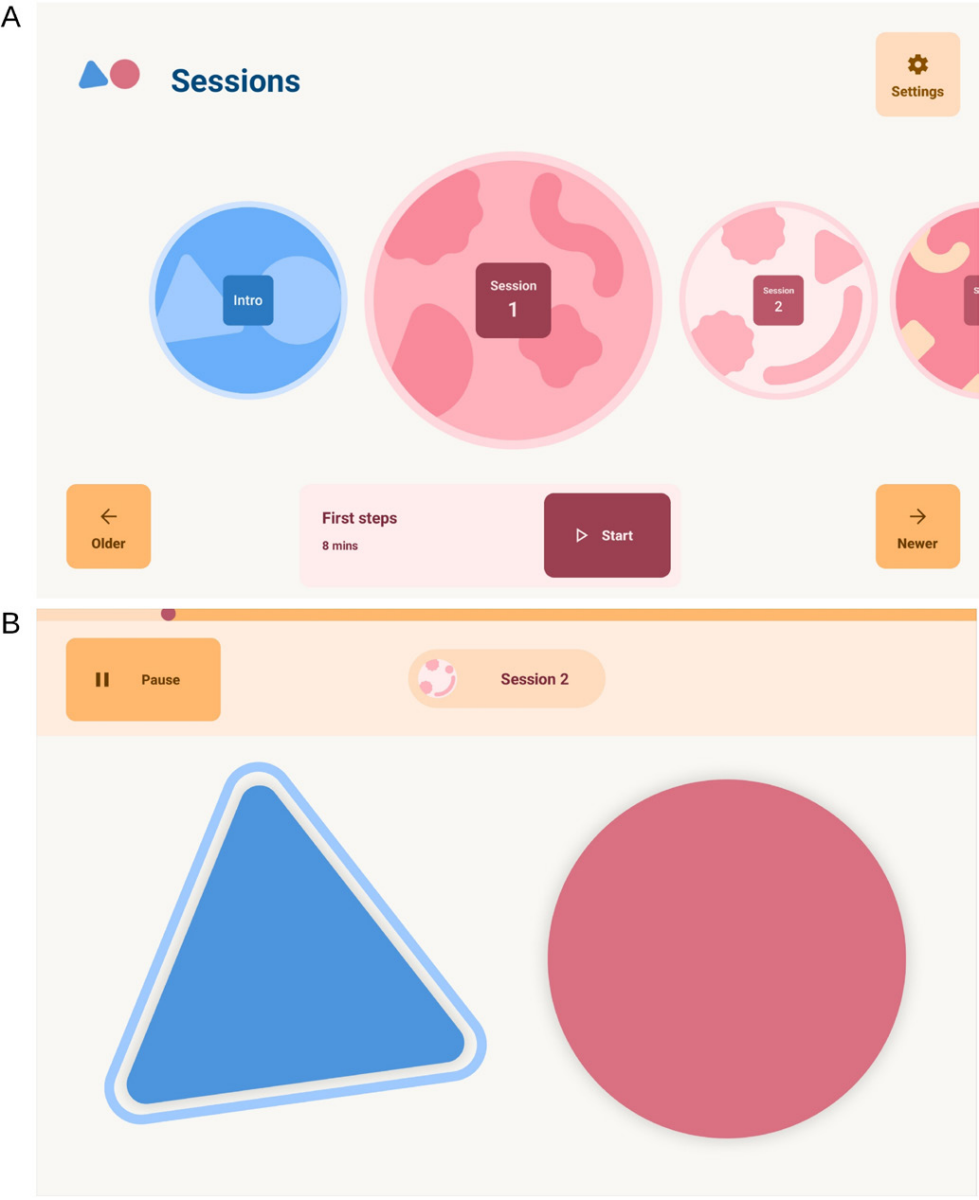
The HD-DRUM app environment. (A) The app home screen interface that allows users to navigate through and select unlocked training sessions. (B) The virtual drums, a blue triangle (high pitch bongo) and red circle (low-pitch bongo), participants use to tap in synchronisation with the audio instructions.

In brief, the app consists of twenty-two ~10–15 min audio training sessions (figure 2A) that introduce rhythmic patterns of different styles including paradiddles, hip-hop, funk and reggaeton with and without a background metronome or music. Participants will be encouraged to drum along with the audio instructions on two virtual drums (figure 2B) on the tablet screen; with their left hand on a blue triangle and with their right hand on a red circle. The virtual drums produce visual (shrinking) and audio feedback (high and low-pitch bongo sounds) when tapped. The patterns gradually increase in complexity and tempo through the programme. Initially, participants practise with each hand separately before using both hands, starting with one hand first and then reversing the order of the hands. Participants will be asked to complete a training session per day, five times a week, for 8 weeks (40 session in total). They will be able to move through the programme at their own pace and repeat each session as often as they wish. Response time and accuracy will be measured within the app and a success threshold of 60%–70% will need to be achieved to move on to the next level. Researchers will remotely assess training adherence by monitoring coded output files generated and uploaded to a project-specific space on Google Firebase (Google) (https://firebase.google.com) when the tablet is connected to the internet.

Participants in the intervention group will be provided with a tablet with the HD-DRUM app installed and an instruction manual for home use. Following completion of baseline assessments participants will receive a face-to-face introduction to the app to familiarise themselves with HD-DRUM. To support retention of the intervention, researchers will maintain weekly contact with participants to provide feedback, assist with any queries or problems and help remove potential barriers. Whenever possible, family members and/or carers will be encouraged to support engagement with the training. Participants will report what support they found helpful in an intervention evaluation questionnaire. Participants in the control condition will be offered the opportunity to borrow a tablet with HD-DRUM to try the training out after they have completed the final assessment of the study.

#### Outcome assessments

##### Primary feasibility outcome assessments

1. Recruitment will be measured as the number of participants assessed for eligibility, eligible, approached and consented to the study. Reasons for not approaching or enrolling eligible participants will be recorded on a screening log.
2. Retention will be measured by the number of participants who complete the study after attending the follow-up visit after 8 weeks. Reasons for withdrawal and loss to follow-up will be recorded where possible.
3. Acceptability will be measured with a semiquantitative self-report questionnaire on rating how easy the training was to use, how engaging it was, appropriateness of frequency, duration and difficulty, perceived beneficial or detrimental training effects, as well as likes, dislikes and potential improvements within the app. This will inform about any further app adjustments required to address the needs of people with HD for a future RCT.
4. Adherence to the training will be automatically tracked within HD-DRUM by recording the frequency and duration of training sessions.

Feasibility success criteria have been predefined according to the following traffic lights system: green (all feasibility rates ≥70%) will signal that the trial was successful, amber (no rates below 40% but at least one below 70%) that the project requires review and design changes and red (at least one rate <40%) that a future RCT will not be feasible.

##### Secondary outcome measures

Performance in the following measures will be assessed before and after 8 weeks of intervention or usual-activity control (see table 1 for participant timeline).

**Table 1:** Schematic diagram of participant timeline

Assessments for all participants			
	Baseline study visit	HD-DRUM training intervention (completed at home with support and reminders) or control time period	Follow-up study visit
	Day 1	8-week intervention period	End of week 8 (+ 2 weeks)
Cohort Descriptive information			
MOCA*	X		
TOPF	X		
Self-report questionnaire			
Patient Health Questionnaire-8	X		X
HD-DRUM participant evaluation survey			X
Paper and pencil tasks			
Stroop task	X		X
Verbal fluency	X		X
SDMT	X		X
Categorical fluency	X		X
Verbal trails (letter-digit switching)	X		X
Computerised tasks			
N-back Audio (letters)	X		X
N-back Visuospatial (square changing positions in grid)	X		X
N-back dual (audio and visuospatial)	X		X
Flanker task	X		X
Q-Motor assessments			
Speeded finger tapping (left/right index finger)	X		X
Finger metronome tapping (left/right index finger)	X		X
Pointing and tapping (dominant hand)	X		X
Dual (pointing and tapping with dominant hand, speeded finger tapping with opposite hand)	X		X
Speeded foot tapping (left/right)	X		X
Metronome foot tapping (left/right)	X		X
Brain imaging			
T1-weighted MPRAGE	X		X
msHARDI	X		X
qMT	X		X
mcDESPOT	X		X
HD-DRUM group			
HD-DRUM training intervention supported by email and telephone reminders		X	
Control group			
Standard care, usual activities		X	

HD, Huntington’s disease; mcDESPOT, multicomponent-driven equilibrium single pulse observation of T1 and T2; MOCA, Montreal Cognitive Assessment; msHARDI, multi-shell high angular resolution diffusion imaging; qMT, quantitative magnetisation transfer; SDMT, Symbol Digit Modalities Test; T1-MPRAGE, T1-weighted Magnetisation Prepared-Rapid Gradient Echo Sequence; TOPF, test of premorbid functioning

### Cognition, motor function and mood

Participants will undergo the following assessments that have been shown sensitive to cognitive^6^ and motor symptoms in HD.^25 26^ The testing session will take approximately 2 hours including breaks.

1. Fine motor functions of speeded finger and foot tapping, tapping in synchronisation with and continuation without a metronome, and dual-task motor ability of simultaneously tapping with one hand and pointing with the other will be assessed with the Quantitative Motor (Q-Motor) assessment test battery.^25^ Mean and SD of tapping and pointing speed will be measured in seconds, mean and SD tapping force will be measured in Newtons.
2. Cognitive functions, notably executive abilities of attention switching, distractor suppression and working memory updating^27^ will be assessed with paper-and-pencil and computerised tasks from the Psychology Experiment Building Language (PEBL) test battery^28^ and implemented in MATLAB.^29 30^ Table 2 provides details about all cognitive tasks including their outcome measures and cognitive domain of interest.
3. Mood will be assessed with the brief self-report Patient Health Questionnaire-8.^31^

**Table 2:** Overview of cognitive assessments

Task	Description	Outcome measures	Targeted domain
Background assessment*			
MOCA^45^	Screening of cognitive functions including visuospatial, attention, executive, memory, naming and orientation abilities.	Number of correct responses with a maximum score of 30	Overall cognitive abilities
TOPF-UK^46^	Participants are instructed to read out a list of 70 phonemically irregular words in ascending order of difficulty.	Number of correctly pronounced words.	Premorbid verbal IQ
Working memory updating			
N-back^47^ auditory	Participants are instructed to press a key when an auditory letter stimulus is identical to N trials back (starting with 1-back, 2-back, etc). Participants complete 5 blocks of 20 trials after 1–2 practice sessions. Task difficulty (N-back level) is varied in response to participants’ performance level measured with the sensitivity index A’(^48^) as follows: If A’ > 0.9 then N+1 If 0.75 >A’ > 0.9 then N remains the same If A’ < 0.75 then N-1 (for blocks of N>1) If A’ < 0.65 in two consecutive 1-back blocks, then discontinue	Maximum N-back level attempted,Sensitivity index A’	Phonological working memory maintenance and updating
N-back visuospatial	Participants respond with a key press when the position of a blue square within a 3×3 grid is the same as N trials back under the same task conditions as above.	Maximum N-back level attempted,Sensitivity index A’	Visuospatial working memory maintenance and updating
Dual N-back (auditory and visuospatial)	Participants perform the auditory and visuospatial N-back tasks simultaneously as described above.	Maximum N-back level attempted,Sensitivity index A’	Working memory maintenance and updating,Attention switching, divided attention
Distractor suppression			
Eriksen Flanker^49^	In each trial (160 in total), a target arrow is displayed in the centre of the screen pointing to the left or right. The target is flanked by arrows pointing in the same direction (congruent) or the opposite (incongruent) direction or by horizontal lines (neutral). Participants are instructed to respond to the target direction by pressing either the right or left shift key and ignore the flanker stimuli.	Reaction times in milliseconds,Accuracy rates for congruent, incongruent and neutral conditions	Interference suppression,Target facilitation
Stroop^50^	Participants are presented with a list of 112 colour words printed in incongruent ink colours. First, they are asked to read out the words (baseline), then to name the ink colour for each word (interference) as accurately and fast as they can within a 2 min time limit.	Number of correct responses, time to complete in seconds for baseline and interference conditions	Interference suppression
Attention switching			
Verbal trails^51^	Participants are asked to count 50 digits upwards and recite 50 letters in alphabetical order (starting with A again when reaching Z) (baseline). They are then asked to switch between counting digits and reciting letters from the starting point 28-D and to switch between reciting letters and counting digits from the starting point R-13 for 25 trials each (50 in total) (switching).	Completion time in seconds,ErrorsSwitching cost (switching–baseline)	Attention switching,Divided attention
Cognitive flexibility			
FAS verbal fluency^52^	Participants are asked to generate as many words as they can starting with the letters F, A and S within a time limit of 1 min per letter.	Number of correct words, repetition and intrusion errors.	Cognitive flexibility and fluency
Category Fluency^52^	Participants are asked to generate as many exemplars from the categories ‘animals’ and ‘supermarket items’ as they can within a time limit of 1 min per category.	Number of correct words, repetition and intrusion errors.	Cognitive flexibility and fluency
Processing speed			
SDMT^53^	Participants are asked to match digits to symbols according to a key given to them by filling in rows of blank boxes underneath the target symbols with the corresponding digits within a time limit of 90 s.	Number of correct responses	Psychomotor speed, visuospatial attention, working memory

* oOnly acquired at baseline for cognitive background information.

A’, alpha prime; MOCA, Montreal Cognitive Assessment; SDMT, Symbol Digit Modality Test; TOPF-UK, Test of Premorbid Functioning–UK edition

### MRI brain morphology and microstructure

MRI data will be collected on a 3 Tesla MRI Siemens Connectom system with ultra-strong (300 mT/m) gradients. Ultra-strong gradients allow the acquisition of diffusion-weighted data with high diffusion-weighting b-values, with good signal-to-noise ratio for the estimation of intracellular diffusion properties.^32^ Multishell high angular resolution diffusion imaging (msHARDI)^33^ data (max b-value=6000 s/mm^2^) will be acquired to estimate microstructural white and grey matter properties of neurite and soma density and soma size.^34 35^ Myelin properties will be estimated with quantitative magnetisation transfer (qMT) imaging^36^ and multicomponent-driven equilibrium single pulse observation of T_1_ and T_2_ (mcDESPOT).^37^ Table 3 provides details of the MRI protocols including their acquisition parameters, the outcome index maps from the analysis based on biophysical modelling, and the estimated white and grey matter tissue properties.

**Table 3:** Overview of MRI data

MRI sequences	Acquisition parameters	Rationale and outcome indices
Magnetisation-prepared rapid gradient-echo (MP-RAGE)	1 mm^3^ resolution, FOV: 256×256, TR=2300 ms, TE=2 ms, TI=857 ms, flip angle: 9; Duration~7 min	3-dimensional (3D) T_1_-weighted anatomical image For the segmentation of regions of interests in the basal ganglia, sensori-motor cortices and cerebellum As reference map for all microstructural outcome maps
Multi-shell High Angular Resolution Diffusion Imaging (msHARDI)^33^	2 mm^3^ resolution; FOV: 220×200; matrix size: 110×110 x 66; TE/TR=59/3000 ms; δ/Δ: 7/24 ms; b-values=0 (14 volumes), 500 (30 directions), 1200 (30 directions), 2400 (60 directions), 4000 (60 directions) and 6000 (60 directions) s/mm^2^; Duration ~20 min	Multishell diffusion weighted imaging data For multitissue constrained spherical deconvolution-based^54^ tractography to reconstruct white matter pathways of interest (corpus callosum, cortico-spinal tract, connections between caudate and prefrontal cortex, connections between putamen and supplementary motor area/motor cortex) For modelling of white matter tissue properties with the Composite Hindered And Restricted Model of Diffusion ^41^ yielding the restricted signal fraction (FR) as an estimate of axon density and the Neurite Orientation Dispersion and Density Imaging^42^ yielding the isotropic signal fraction as an estimate of free water and the orientation dispersion index as an estimate of axon orientation and dispersion. for modelling of grey matter tissue properties with the Soma And Neurite Density Imaging model^35^ yielding estimates of soma density (soma signal fraction) and size.
Quantitative magnetisation transfer (qMT)^55 56^	1.72 mm^3^ resolution; FOV 220×220×179 mm; matrix size 128×128 x 104; turbo flash sequence, turbo factor 4, non-selective excitation MT pulse duration: 15.3 ms, 11 MT-weighted volumes and 1 vol without MT-weighting, 11 Frequency offsets (Hz) and 11 flip angles (degrees): 47 180 (628°); 56 360 (332°); 12060, (628°); 1000 (332°);1000 (333°); 2750 (628°); 2770 (628°); 2790 (628°); 2890 (628°); 1000 (628°); 1000 (628°); Duration ~25 min	qMT data to model the macromolecular proton fraction as an estimate of white matter myelin content^36^.
Multicomponent-driven equilibrium single pulse observation of T_1_ and T_2_ (mcDESPOT)^37^	1.72 mm^3^ resolution; FOV 220×220×179 mm; matrix size 128×128×104;T1-weighted 3D spoiled gradient-recalled echo sequence (SPGR) with TR=4 ms; TE=1.9 ms; 8 flip angles (3°, 4°, 5°, 6°, 7°, 9°, 13° and 18°); Inversion recovery-prepped spoiled gradient-recalled echo sequence with TR=4 ms; TE=1.9 ms; flip angle 5°; steady-state free precession with TR=4.54 ms, TE=2.27 ms, 8 flip angles (10°, 13.33°, 16.67°, 20°, 23.33°, 30°, 43.33° and 60°), duration ~14 min	mcDespot data to model The myelin water fraction as an estimate of white matter myelin To estimate T_1_ and B_1_ fields for qMT analyses

FOV, field of view; mcDESPOT, Multicomponent Driven Equilibrium Single Pulse Observation of T1 and T2; MT, magnetisation transfer; TE, echo time; TI, inversion time; TR, repetition time

All MRI protocols have been piloted in individuals with HD. They take 60 min in total to acquire and are well tolerated. Our previous research has identified microstructural differences in HD patients compared with healthy controls.^38 39^ Importantly, we found drumming training-induced increases in the qMT-based macromolecular proton fraction in callosal WM suggestive of increased myelin.^19^

### Sample size

Following the Consolidated Standards of Reporting Trials (CONSORT) statement extension to randomised pilot and feasibility trials,^40^ no formal power calculations have been conducted. We aim to recruit 50 individuals with HD. This number is pragmatic given the size of the target population, level of engagement required and resources available. It will allow estimation of recruitment, retention and adherence rates with a 95% binomial CI of no more than plus or minus 15 percentage points irrespective of the point estimate.

The combined population of the participating patient identification centres (PICs) should be adequate to achieve recruitment target, but this assumption will be tested as part of the feasibility assessment. While there is facility to add more PICs, further strategies to enhance recruitment will not be used within this feasibility trial.

### Assignment of interventions

#### Allocation

Randomisation will be stratified for TFC score to ensure comparability of the groups with regard to disease burden. There will be five strata corresponding to TFC scores of 9, 10, 11, 12 and 13, respectively. One randomised group allocation sequence per stratum will be computer generated based on pseudo-random numbers by a statistician (PP) independent of patient recruitment and data collection, using R V.4.1.0^41^ and uploaded onto the HD-DRUM project-specific Research Electronic Data Capture (REDCap)^42^ site.

#### Blinding (masking)

The researchers conducting the baseline assessments (cognitive, motor and MRI) have no access to the randomisation list implemented in REDCap and will be blind to the group allocation until baseline assessments are completed. However, once group allocation has taken place at the end of the baseline assessments, blinding of the researchers involved in the data collection and participants will not be possible due to the nature of the intervention and control conditions and due to limited resources and staffing. The analysis of the Q-Motor data will be conducted blind to group allocation by an independent researcher not involved in the data collection (RS). The lack of blinding of the clinical and MRI outcomes is a limitation of the study design, but quantitative outcome measures have been used where possible to minimise any potential biases that may be introduced due to a lack of blinding.

### Data collection, management and analysis

#### Data collection methods

The project will be conducted following Good Clinical Practice, data protection in accordance with the Data Protection Act 2018, and CUBRIC standard operating procedures (SOPs) including MRI safety and operation guidelines. The project will adhere to any applicable Centre for Trials Research SOPs, including those for project management, data management and protection, and adverse/serious adverse event reporting. Research staff will receive training in the above.

Training-specific performance improvements, namely speed and accuracy of drumming, will be recorded by the HD-DRUM app. Frequency and duration of training engagement will also be recorded by HD-DRUM as a measure of adherence.

Cognitive and motor performance measures will be collected on paper data collection forms and electronically via PEBL and MATLAB on a personal computer in a quiet testing room in CUBRIC. Electronic data capture of Q-Motor data are automated by the Q-Motor data acquisition software Q-MedX.

MRI data will be acquired on the 3 Tesla MRI Siemens Connectom system at CUBRIC.

#### Data management

Participants will be assigned a study and CUBRIC ID and all electronic and hard copy data will be coded with these IDs. Electronic data will be stored within Cardiff University’s firewall and password-protected computer systems. Cognitive and motor clinical outcome and training data (stored in .csv files) will be managed with the REDCap web application (www.project-redcap.org) that allows secure storage, management and sharing of data using a confidential security level. Paper record forms will be scanned, and electronic copies will be uploaded onto RedCAP. Cognitive and motor scores are entered by the researcher (VI) and will be quality-checked against the original record forms by other members of the research team.

The data collection with the drumming training application complies with General Data Protection Regulation (GDPR) and data privacy regulations. No personal or identifiable user information is stored on the tablet or on Google Firebase. The drumming training output files are coded with a sixteen-digit-letter-string tablet ID. Firebase services encrypt data at rest and during transit using hypertext transfer protocol secure.

MRI data will be stored securely on the CUBRIC partition of XNAT (www.central.xnat.org). Study and CUBRIC ID keys and any personal information will be stored for the duration of the project securely in a locked cupboard in the principal investigator’s (CM-B) office in CUBRIC. Only researchers associated with the study will have confidential access to files, which allow the matching of recorded data to participants. No data, whether paper or electronic, will leave Cardiff University sites without being completely coded, that is, all identifiable data will be removed from the dataset. Any electronic files or disks will be stored on Cardiff University sites and electronically on Cardiff University systems. At the conclusion of the study, participant-identifiable data will be destroyed and non-identifiable data will be archived, although it will still be accessible to the study team. Data will be archived for 15 years, in line with Cardiff University policies and procedures.

### Statistical methods

The study will be reported in accordance with the CONSORT statement extension to randomised pilot and feasibility trials.^40^ As this is a feasibility trial, it is not formally powered to test for effectiveness of the intervention while controlling type I error. The primary aim is to assess the feasibility. For this purpose, recruitment, retention and adherence rates and acceptability rating scores and thematic responses will be documented and evaluated according to the success criteria described above. Feasibility percentage rates will be calculated as follows:

- Recruitment rate=100×(number of participants consented/number of participants eligible and approached) %.
- Retention rate=100×(number of participants completing the study at 8-week follow-up/number of participants consented) %.
- Adherence rate (frequency)=100×(number of days app used for training/40 days) %.
- Adherence rate (duration)=100×(number of minutes app used for training /(40 days×11.3 min average session duration=451 min) %.

Descriptive summaries such as mean and SD estimates (for continuous variables) of effect sizes and 95% CIs will be calculated for all secondary clinical and MRI outcome measures. Motor and mood measurements are detailed in the outcome section above and information about the cognitive and MRI outcome measures can be found in tables2  3. Differences in mean and SD of participants’ absolute and percentage changes from baseline will be calculated to provide estimates of effect size and variability of any performance changes in the two groups. All calculations will be conducted in SPSS^43^ and R.^41^ This will provide information to help estimate the sample size required for a future definitive RCT into HD-DRUM in this patient population. Note that the absence of a change in individuals with HD in the HD-DRUM group compared with the control group may suggest a beneficial effect of the intervention as HD is a progressive neurodegenerative disease.

### Monitoring

#### Data monitoring and ethics oversight

This feasibility trial is expected to pose minimal risk to participants, and hence no formal data monitoring committee has been established. However, the research team will produce regular data monitoring reports for the project steering group, composed of a trial statistician, clinician and public involvement member, who are independent of the project management team and will assess and monitor the data quality on an annual basis throughout the study.

#### Harms

MRI scanning is non-invasive and has no known significant adverse health effects when appropriate screening and safety measures are in place and implemented. CUBRIC SOPs will be followed to ensure this. Any adverse effects including rare events of peripheral nerve stimulation, dizziness, mild nausea during the MRI scanning will be monitored, recorded and documented. These side effects, if experienced, may be uncomfortable but resolve after leaving the magnetic field and participants will be warned at the beginning of the scanning session. It will also be explained that the researchers do not have expertise in MRI diagnosis, as they are not medical doctors. Participants should not regard the research scans as medical screening procedures and if they had any health concerns, they should contact their general practitioner (GP). In the unlikely event of an unexpected finding, a neurological consultant (AER) will be asked to examine the scans, and if appropriate to report back to the participant’s GP.

Finally, in the unlikely event of adverse events occurring from the HD-DRUM intervention or the cognitive and motor assessments, these will be monitored, recorded and reported.

## Ethics and dissemination

### Research ethics approval

The study will be conducted in accordance with the recommendations for physicians involved in research on human participants adopted by the 18th World Medical Assembly, Helsinki 1964 and later revisions. Ethical approval for this feasibility study was given by the Wales Research Ethics Committee (REC) 2 (22/WA/0147). Cardiff University has agreed to act as a sponsor for the study (SPON1895-22).

All amendments to the ethical approval will be recorded in the trial registration and any future amendments to be made will be done in accordance with the Integrated Research Application System regulation and will be communicated to sites via the UK permissions system.

### Consent

Informed written consent will be sought by means of participants’ dated signature and dated signature of the person who obtained the informed consent on the consent form provided in online supplemental appendix. The participant will be allowed as much time as needed to consider the PIS and given the opportunity to ask the researcher or independent parties any questions to decide on participation. All participants will have the procedures explained to them in detail, including that the study design is randomised, and be reminded that they can withdraw at any time, without the need to give a reason. The original signed consent form will be retained at the study site in CUBRIC, and participants will receive a copy.

### Access to data

This project has been endorsed by the Enroll-HD Scientific Oversight Committee (SOC) (14/11/2022) and use of Enrol captured data has been approved for this trial. Enrol captured UHDRS data together with volumetric measurements of the caudate and putamen from the T_1_ weighted images acquired in this study, will allow us to report our outcome measures stratified according to the new Huntington’s Disease Integrated Staging System.^44^ ENROLL-HD captured data will also allow us to explore the impact of clinical symptoms including depression and apathy on feasibility. At the end of the project, the coded study data will be shared and made accessible to the research community *via* the Enroll-HD-specific data request process.

### Dissemination policy

Dissemination activities to share the progress and results of our projects with people affected by HD will be led by our PPI contributors. Dissemination strategies will include information sessions for people affected by HD, clinicians, and the general public, as well as communications *via* social media, newsletters and websites of the HDA, the European Huntington Disease Network (EHDN) and HCRW. The findings of this trial will be disseminated as scientific publications and presentations at conferences including at the biennial EHDN plenary meeting. Authorship eligibility will be assessed following the International Committee of Medical Journal Editors guidelines.

## supplementary material

10.1136/bmjopen-2023-082161 online supplemental file 1
