# Supplementary material for: Protocol for a randomised controlled unblinded feasibility trial of HD-DRUM: a rhythmic movement training application for cognitive and motor symptoms in people with Huntington’s disease
Source: BMJ Open. 2024 Jul 31;14(7):e082161. doi: 10.1136/bmjopen-2023-082161 (PMC11418498; doi:10.1136/bmjopen-2023-082161)
Supplement: online supplemental file 1 [file bmjopen-14-7-s001.pdf]

HD PATIENT PARTICIPANT CONSENT FORM

Study title: Feasibility randomised controlled pilot study of HD-DRUM - a novel motor sequence training app for people with Huntington's disease.

Chief Investigator: Dr Claudia Metzler-Baddeley, **Sponsor**: Cardiff University

Please initial each box and sign at the end

1. I confirm that I have read and understand the information sheet dated [ ] (version 1.7) for the above study that will involve assessments of cognitive and motor functions and MRI brain scanning before and after 2 months of digital drumming training or non-intervention control. I understand that if I am not eligible for the MRI scan, I can continue with the cognitive assessments and the training (or control). I have had the opportunity to consider the information and ask questions, which have been answered satisfactorily. ☐
2. I understand that my participation is voluntary and that I am free to withdraw at any time, without giving a reason, and understand that my medical care or legal rights will not be affected. Data collected up until my withdrawal may still be used in the study. ☐
3. I understand that relevant sections of my medical notes, and data collected during the study, may be looked at by individuals from the Cardiff University research team and from the regulatory authorities. I give permission for these individuals to have access to the relevant parts of my records. ☐
4. I understand and agree that the MRI scan is not a medical screening procedure and that the researchers are not qualified to provide a clinical diagnosis or identify potential abnormalities. If the researchers are concerned that there may be a potential abnormality on the scan, I consent to them disclosing the scan to a specialist neuroradiologist to provide a radiological report on the scan and to report to my GP. ☐
5. I understand the risks of MRI and that if I misrepresent my health to the researchers including failure to disclose presence of indwelling metallic devices/clips, the study may put me at risk and be potentially harmful to me. ☐

☐

6. I understand that I will be randomly allocated to either the drumming training intervention or the non-intervention control group so that the groups are balanced for sex, age, and disease stage. If I am allocated to the control group, I will have the opportunity to engage with the drumming training after completion of the study.

7. If I am allocated to the drumming training group, I agree for my drumming training to be digitally recorded. These will be used as part of the study assessment. ☐

8. I agree for my drumming performance to be video recorded before and after the training. ☐

9. I agree for my responses in a task of coming up with words beginning with a specific letter (e.g. A) or things of a specific category (e.g. animals) to be audio recorded to help the researchers scoring my responses. I understand that these recordings will be deleted after my responses have been scored. ☐

10. I consent to my family members, friends, or carers being approached as healthy control participants for the study. ☐

11. I consent for quotations of my feedback to the training to be published in an anonymised form. ☐

12. I understand that the information provided by me will be held confidentially. Data from all parts of this study will be stored using a code so that only the researchers can trace this information back to me individually (coded-linked). I understand that any information that could identify me will be kept strictly confidential and that no personal information will be included in the study report or other publication. The information will be retained securely for a minimum of 15 years in line with Cardiff University's policies. ☐

13. I consent to the coded-linked data obtained from all parts of this study being shared with other researchers at Cardiff University or at external institutions for research purposes only. ☐

14. I consent to being contacted about participating in further research linked to this study. ☐

15. I understand that HD-Drum is recruiting participants who are already part of a research study named "Enroll-HD". Enroll-HD collects clinical information about you and your health as well as biological samples. HD-Drum will request access to the information collected about you during your participation in the Enroll-HD study. The Enroll-HD information will be used to ☐

understand and analyze the information collected about you during HD-Drum. If you are not already participating in Enroll-HD but would like to join this study, please speak to your study site team [ ].

16. I agree to take part in the above study. ☐

______________________	________________	____________________
Name of Participant	Date	Signature
______________________	________________	____________________
Name of Researcher	Date	Signature

1 copy for the file, 1 copy for the participant
