# Supplementary material for: Protocol for a randomised controlled unblinded feasibility trial of HD-DRUM: a rhythmic movement training application for cognitive and motor symptoms in people with Huntington’s disease
Source: BMJ Open. 2024 Jul 31;14(7):e082161. doi: 10.1136/bmjopen-2023-082161 (PMC11418498; doi:10.1136/bmjopen-2023-082161)
Supplement: Supplementary file 2 [file bmjopen-2023-082161.reviewer_comments.pdf]

PEER REVIEW HISTORY

BMJ Open publishes all reviews undertaken for accepted manuscripts. Reviewers are asked to complete a checklist review form (**http://bmjopen.bmj.com/site/about/resources/checklist.pdf**) and are provided with free text boxes to elaborate on their assessment. These free text comments are reproduced below.

ARTICLE DETAILS

**TITLE (PROVISIONAL)**	Protocol for a randomised controlled unblinded feasibility trial of HD-DRUM, a rhythmic movement training application for cognitive and motor symptoms in people with Huntington's disease.
**AUTHORS**	Ioakeimidis, Vasileios; Busse, Monica; Drew, Cheney J. G.; Pallmann, Philip; Watson, Guy; Jones, Derek; Palombo, Marco; Schubert, Robin; Rosser, Anne E.; Metzler-Baddeley, Claudia

VERSION 1 – REVIEW

**REVIEWER**	McCusker, Elizabeth<br>Westmead Hospital and Sydney Medical School University of Sydney, Neurology Dept
**REVIEW RETURNED**	15-Jan-2024

**GENERAL COMMENTS**	Comments on and suggestions for the proposal by Vasileios et al Protocol for a randomised controlled feasibility trial of HDDRUM, a rhythmic movement training application for cognitive and motor symptoms in people with Huntington's disease. The research tool to be evaluated in an RCT is the HD-DRUM app, a computerised application of the drumming training intervention.<br>The investigators make a plausible case that the intervention has a potential to improve both motor and cognitive function.<br>The following comments are suggestions only for consideration but made with a larger RCT in mind.<br>The primary goal of this proposal is 'To assess the feasibility of a larger effectiveness RCT investigating eight-weeks of at-home HD-DRUM intervention compared with usual activities in people with HD.' That this RCT is unblinded needs to be included in the title and the abstract.<br>Of the secondary goals, two should have priority. Firstly, the essential measures of feasibility; recruitment, retention, accessibility, and adherence and add reliability are key outcomes. In addition, the goal to determine 'Performance measures in cognitive and motor tasks for sample size calculations for a future RCT' are required for planning a larger trial.<br>Another secondary goal of MRI imaging with unblinded assessment is likely an expensive and time- consuming part of the study for participants and researchers. This secondary goal to determine MRI change will be affected significantly if the primary goal is not achieved. Eight weeks is a short time frame to expect meaningful changes.<br>The previous study conclusion of 'Importantly, we found drumming training-induced increases in the qMT-based macromolecular

	proton fraction (MPF) in callosal white matter suggestive of increased myelin19', (page 16 lines 8-10), is noted. However, a case can be made that it would be better to establish the primary and priority secondary goals before undertaking the imaging component.<br>This is particularly as the researchers state that there are limitations to the primary goal of low participant numbers and unblinding of clinical and MRI assessments due to budgetary constraints. This is overcome in part by the inclusion of objective measures such as the Q motor assessment but will affect the validity of results.<br>The inclusion criteria are too wide for such a small number of participants. There is no upper age limit or disease range CAG limit that could be better represented by addition of a CAP score. The TFC has some validation but the use of that alone (Allocation: 'There will be seven strata corresponding to TFC scores of 9, 10, 11, 12 and 13, respectively) for staging by a difference of only one point needs to be replaced by the carefully researched, Huntington's Disease Integrated Staging System (HD-ISS) * developed for research stages earlier in the disease course. HD-ISS includes the UHDRS Independence scale score (100-0) with more subtle gradations of function for premanifest to manifest HD. The Total motor score (TMS) is also essential to identify the early motor signs that may not have motor symptoms e.g. eye movement changes, subtle chorea and minor finger tap impairment.<br>'Usual activity' in the control group is not defined but could vary significantly e.g. some of whom may undertake activities which enhance motor skills such as playing a musical instrument, regular computer use or participation in sports.<br>Whilst it is valid to state in the Abstract and Introduction first sentences that 'progressive cognitive and motor decline, (is) largely due to basal ganglia (BG) atrophy' there is other wider pathology with significant impact relevant to the study in prefrontal regions and in fronto-striatal connections as referenced (ref 3 and 27).<br>A particular issue to address in this protocol that could impact the primary outcome is that of apathy which is a more prominent feature of early HD and throughout than depression **. Has the PHQ-9 been validated in Huntington disease against companion report?<br>For an at home study, it would be important to have a companion/carer input and opinion about mood and motivation as well as support to engage in the study and for inclusion in a future larger study.<br>A witness to the participant's signature other than the researcher needs inclusion on the consent form.<br>* Tabrizi SJ, Schobel S, Gantman EC, et al ; Huntington's Disease Regulatory Science<br>Consortium (HD-RSC). A biological classification of Huntington's disease: the<br>Integrated Staging System. Lancet Neurol. 2022 Jul;21(7):632-644.<br>**Connors MH, Teixeira-Pinto A, Loy CT. Apathy and Depression in Huntington's<br>Disease: Distinct Longitudinal Trajectories and Clinical Correlates.

	J<br>Neuropsychiatry Clin Neurosci. 2023 Winter;35(1):69-76.

**REVIEWER**	Kulisevsky, Jaime<br>Movement Disorders Unit, Neurology Department, Hospital de la Santa Creu i Sant Pau, Universitat Autònoma de Barcelona
**REVIEW RETURNED**	23-Jan-2024

**GENERAL COMMENTS**	The authors present a feasibility protocol for a non-pharmacological treatment based on a rhythmic movement training app for motor and cognitive symptoms of Huntington's disease (HD). The treatment is based on a specific technique of neurological music therapy that utilizes rhythms to trigger movements in an attempt to regulate the loss of rhythmic timing signals generated by dysfunction in cortico-subcortical basal ganglia circuits. The proposed design by the authors seems appropriate but perhaps overly ambitious for an 8-week feasibility study. Additionally, I believe the authors should reconsider certain aspects of the protocol that could benefit them. My comments are as follows:<br><br>According to the inclusion criteria, participants in the presymptomatic stage (PreHD) are allowed entry. What is the justification for including them? It is not justified to assess the effectiveness of an intervention on clinical symptoms in an individual in the PreHD stage, therefore without symptoms.<br><br>In the same vein, the inclusion criteria allow entry for participants with a Total Functional Capacity (TFC) of 9-13. Symptomatic treatments should be focused on patients with the corresponding clinical manifestations that are aimed to treat. A patient with a TFC of 13 implies that he/she can lead a completely normal life because they are functionally preserved. I understand the justification from a neuroimaging perspective, as it is well known that micro and macrostructural changes already exist years before the clinical diagnosis of HD. However, including patients without a minimum level of cognitive and motor symptoms may introduce bias in primary and secondary outcomes.<br><br>One of the inclusion criteria should consider a specific range for CAG repetitions. Including patients at extreme ranges with few or many repetitions can introduce significant bias in the data.<br><br>I understand the rationale for conducting this feasibility study for future randomized clinical trials, but I think it has a design that is too ambitious for a feasibility study. A randomized clinical trial of longer duration could be conducted perfectly well. Have the authors considered the possibility of calculating the sample size based on a validated cognitive instrument for HD? I would suggest the Parkinson's Disease Cognitive Rating Scale (PD-CRS), which has already been validated as a cognitive monitoring instrument and has a sufficient score range to calculate reasonable sample sizes.<br><br>Arnau Puig-Davi PhD, helped with the present review.

VERSION 1 – AUTHOR RESPONSE

Ad Reviewer 1:

The primary goal of this proposal is 'To assess the feasibility of a larger effectiveness RCT investigating eight-weeks of at-home HD-DRUM intervention compared with usual activities in people with HD.' That this RCT is unblinded needs to be included in the title and the abstract.

The title and the abstract now include that the RCT is unblinded.

Of the secondary goals, two should have priority. Firstly, the essential measures of feasibility; recruitment, retention, accessibility, and adherence and add reliability are key outcomes.

The primary objective of this trial is the assessment of the feasibility of a larger effectiveness RCT investigating eight-weeks of at-home HD-DRUM intervention compared with usual activities in people with HD (please see primary objective on page 7). The primary feasibility outcome measures of recruitment, retention, accessibility, and adherence are described on pages 12/13.

We were unsure about what the reviewer meant with adding reliability. Would this be reliability of the HD-DRUM intervention? Please note that the HD-DRUM app was co-designed and developed with people with HD and we have published the development process https://doi.org/10.2196/48395. Please also note that we will assess the reliability of the app in terms of ease of use with a semi-quantitative self-report evaluation questionnaire as part of our acceptability assessments (page 13). The data capture of the app will allow us to assess the reliability of measuring performance changes with the app.

Another secondary goal of MRI imaging with unblinded assessment is likely an expensive and time-consuming part of the study for participants and researchers. This secondary goal to determine MRI change will be affected significantly if the primary goal is not achieved. Eight weeks is a short time frame to expect meaningful changes. The previous study conclusion of 'Importantly, we found drumming training-induced increases in the qMT-based macromolecular proton fraction (MPF) in callosal white matter suggestive of increased myelin19', (page 16 lines 8-10), is noted. However, a case can be made that it would be better to establish the primary and priority secondary goals before undertaking the imaging component.

To exploit fully the potential therapeutic benefits of HD-DRUM and for the purpose of personalizing the training, it is necessary to gain a mechanistic understanding of the effects of HD-DRUM on the brain. We have secured external funding from National Institute of Health and Care Research (NIHR) and Health and Care Research Wales (HCRW) and have gained ethical approval for the here proposed MRI assessments which will allow the investigation of neural mechanisms that may underpin any training benefits including myelin remodelling and synaptic sprouting. Importantly, MRI assessments to quantify caudate and putamen volumes will be required to implement the new HD-ISS staging system in a future RCT (see point below). Thus, MRI assessments will be an important part of a future RCT into efficacy and mechanisms, and hence require feasibility assessment in terms of acceptability and the likelihood of capturing suitable quality images for analysis as movement artefacts are a known problem in this population. For this purpose, we are collaborating with

physicists colleagues in CUBRIC who are developing techniques for the correction of motion artefacts in T1 weighted raw data required for volumetric analyses.

There is ample evidence demonstrating that changes in the here proposed MRI measurements that are sensitive to microstructural properties of brain tissue including neurite density and myelin can be detected

after relative short periods of training. We chose 8 weeks because we have previously found microstructural MRI changes after 8 weeks of drumming training in HD (https://doi.org/10.3233/jhd-200424, https://doi.org/10.3233/jhd-140113) as well as after 8 weeks of working memory training in healthy participants (https://doi.org/10.1162/jocn_a_01127, https://doi.org/10.1523/jneurosci.1973-15.2016). These findings accord with other human imaging training studies (e.g. microstructural changes after 6 weeks of complex motor skill learning https://doi.org/10.1038%2Fnn.2412) and with animal plasticity studies that have detected myelin remodelling after a few hours or days of motor skill learning (https://doi.org/10.1523/jneurosci.3048-13.2013, https://doi.org/10.1126/science.1254960). Thus, we would argue that there is sufficient evidence justifying the assumption that meaningful changes in microstructural MRI measurements can be expected after 8 weeks.

This is particularly as the researchers state that there are limitations to the primary goal of low participant numbers and unblinding of clinical and MRI assessments due to budgetary constraints. This is overcome in part by the inclusion of objective measures such as the Q motor assessment but will affect the validity of results.

We agree that unblinding due to the nature of the intervention will affect the validity of the study and this is mentioned in the Strengths and Limitation and blinding sections. However, we will attempt to limit any confounding effects of unblinding by randomizing participants into groups only after completion of all baseline assessments, by employing objective computerized assessments of cognitive and motor functions, and by independent analyses of the Q-motor assessments blinded to groups.

With regards to the participant numbers, it is noteworthy that this is a feasibility study for a future RCT and as such no formal power calculation was conducted. The number of 50 participants is pragmatic and commensurate with the size of similar studies and the recommended sample size for feasibility trials (https://doi.org/10.1097%2FMLR.0000000000001664).

The inclusion criteria are too wide for such a small number of participants. There is no upper age limit or disease range CAG limit that could be better represented by addition of a CAP score.

Following NIHR and HCRW guidelines (NIHR-INCLUDE https://sites.google.com/nihr.ac.uk/include/home/guidance) our study aims to be as inclusive as possible and this was noted by the review panel as a commendable aspect of the study. The aim of the study is to gain feasibility information from an as wide as possible range of participants so that informed decisions about inclusion and exclusion criteria for a future RCT can be made.

*The TFC has some validation but the use of that alone (Allocation: 'There will be seven strata corresponding to TFC scores of 9, 10, 11, 12 and 13, respectively) for staging by a difference of only one point needs to be replaced by the carefully researched, Huntington's Disease Integrated Staging System (HD-ISS) * developed for research stages earlier in the disease course. HD-ISS includes the UHDRS Independence scale score (100-0) with more subtle gradations of function for premanifest to manifest HD. The Total motor score (TMS) is also essential to identify the early motor signs that may not have motor symptoms e.g. eye movement changes, subtle chorea and minor finger tap impairment.*

The HD-ISS staging system was not published when this study protocol was developed and funding was applied for, and our understanding is that it is not fully operationalized yet. Classification into stages 0-2 requires quantification of caudate and putamen volumes from T1 weighted MRI scans. This information is not routinely collected in UK HD clinics and hence cannot be provided to us by our participating recruiting clinics, nor do we have the funds to conduct screening MRI scanning prior to enrolment into the study for the purpose of participant staging and randomization.

However, the MRI aspect of our study will allow us to report the outcome data of this study according to the HD-ISS staging system because we can use the baseline T1 weighted MRI data to perform volumetric analysis of the basal ganglia. This has been added to the manuscript on page 24 which also explains that the study has been endorsed by ENROLL-HD and that we will be able to gain access to participants' UHDRS data.

We have corrected the number of strata to five in the manuscript (page 16).

'Usual activity' in the control group is not defined but could vary significantly e.g. some of whom may undertake activities which enhance motor skills such as playing a musical instrument, regular computer use or participation in sports.

We agree that usual activities may vary significantly between participants. However, given that we randomise participants into the two groups, we expect the incidence of these confounding variables to be as frequent in the control as in the intervention arm.

Whilst it is valid to state in the Abstract and Introduction first sentences that 'progressive cognitive and motor decline, (is) largely due to basal ganglia (BG) atrophy' there is other wider pathology with significant impact relevant to the study in prefrontal regions and in fronto-striatal connections as referenced (ref 3 and 27).

This has been added to the introduction on page 5.

*A particular issue to address in this protocol that could impact the primary outcome is that of apathy which is a more prominent feature of early HD and throughout than depression **.*

We agree that apathy may potentially affect recruitment and retention. We will have access to Enroll-HD captured data about apathy from the Problem Behaviours Assessment which will allow us to explore whether apathy had an effect on feasibility in term of recruitment, adherence, and retention. This has been added to page 24.

Has the PHQ-9 been validated in Huntington disease against companion report?

The PHQ-9 is a widely applied brief screening tool for depression that has been used in HD https://doi.org/10.3390/brainsci12020161. We initially planned to use the Hamilton Depression Scale for
mood assessment but decided to switch to the shorter PHQ-8 (which excludes one question about suicidal thoughts) to reduce participants' burden.

For an at home study, it would be important to have a companion/carer input and opinion about mood and motivation as well as support to engage in the study and for inclusion in a future larger study.

We agree that family and carers' support to engage in the study is important. Whenever possible, family members and/or carers will be encouraged to support the participant with the training. Participants will also be asked about what support they found helpful in an intervention evaluation questionnaire at the end of the study. This information has been added on page 12.

A witness to the participant's signature other than the researcher needs inclusion on the consent form.

We respectfully disagree with this point as only people with capacity to consent are included in this study and our ethical approval does not require a witness to the participant's signature other than the researcher.

Ad Reviewer 2:

According to the inclusion criteria, participants in the presymptomatic stage (PreHD) are allowed entry. What is the justification for including them? It is not justified to assess the effectiveness of an intervention on clinical symptoms in an individual in the PreHD stage, therefore without symptoms.

There are a number of reasons for including individuals at PreHD stage: firstly, atrophy of the caudate and putamen and white matter degeneration as well as subtle cognitive problems (notably executive

dysfunction) and changes in mood are already present at PreHD stage and may benefit from the training. Secondly, we assume that the HD-DRUM training would be most beneficial in terms of

maintaining function and/or slowing disease progression if started as early as possible to build up resilience in brain networks. Thirdly our pilot research suggested that the training was most suitable for people at PreHD and/or early stages of the disease.

In the same vein, the inclusion criteria allow entry for participants with a Total Functional Capacity (TFC) of 9-13. Symptomatic treatments should be focused on patients with the corresponding clinical manifestations that are aimed to treat. A patient with a TFC of 13 implies that he/she can lead a completely normal life because they are functionally preserved. I understand the justification from a neuroimaging perspective, as it is well known that micro and macrostructural changes already exist years before the clinical diagnosis of HD. However, including patients without a minimum level of cognitive and motor symptoms may introduce bias in primary and secondary outcomes.

Please see our response outlining the rational for including individuals with PreHD above. Caudate atrophy and subtle cognitive impairment and mood disorder are well-established in PreHD many years before the onset of motor symptoms and the objective of HD-DRUM is to address these problems. The longer-term
objective of the training is to see if disease progression can be delayed, assuming that the earlier the training can commence the larger any benefits. We would not expect the training to reverse any neurodegeneration but potentially delay the disease progression.

This study has been reviewed by the Enroll-HD Scientific Oversight Committee. Based on our pilot data suggesting that the training was most suitable for individuals at earlier disease stages, they recommended to include participants with TFC between 9-13. We will be able to explore the primary and secondary outcome data in terms of the impact of disease burden on effect sizes.

One of the inclusion criteria should consider a specific range for CAG repetitions. Including patients at extreme ranges with few or many repetitions can introduce significant bias in the data.

We are collecting information about CAG repetitions and will use this information to describe the cohort. If individuals with extreme CAG values are present, we estimate and report any bias in our primary and secondary outcome measures.

I understand the rationale for conducting this feasibility study for future randomized clinical trials, but I think it has a design that is too ambitious for a feasibility study. A randomized clinical trial of longer duration could be conducted perfectly well.

The objective of this feasibility trial is to gather information about several unknown aspects of the intervention that would need to be clarified before embarking on an expensive fully-powered RCT.

For instance, it remains unknown whether it is possible to recruit into such a non-pharmacological complex intervention trial in the environment of competing pharmacological, disease-modifying studies; whether participants will adhere to the intervention; whether they find the intervention acceptable; what the most sensitive clinical and MRI outcome measures are to capture training-induced changes in a future RCT etc.

Have the authors considered the possibility of calculating the sample size based on a validated cognitive instrument for HD? I would suggest the Parkinson's Disease Cognitive Rating Scale (PD-CRS), which has already been validated as a cognitive monitoring instrument and has a sufficient score range to calculate reasonable sample sizes.

As this is a feasibility study with the primary objective of estimating rates of recruitment, retention, adherence and acceptability, no formal sample calculation is required; the sample size was determined in line with recommendations for feasibility trials (https://doi.org/10.1097%2FMLR.0000000000001664) and the CONSORT extension to randomized feasibility trials which states that hypothesis testing is not indicated (https://doi.org/10.1136/bmj.i5239).

However, as the study has been endorsed by Enroll-HD, we will be able to gain access to data from the United Huntington's disease rating scale (UHDRS) including cognitive data and will be able to explore effect sizes on UHDRS cognitive measurements for apriori sample size calculation for a future RCT.

VERSION 2 – REVIEW

**REVIEWER**	McCusker, Elizabeth<br>Westmead Hospital and Sydney Medical School University of Sydney, Neurology Dept
**REVIEW RETURNED**	15-Mar-2024

**GENERAL COMMENTS**	Re review BMJ Open HD DRUM<br>The authors address some points raised by this reviewer. However, a number remain unresolved in this reviewer's opinion, despite the rebuttal. In summary, unblinded assessments and wide inclusion criteria and reliance on TFC.<br>As well as all the other stated aims, reliability for this purpose means whether this app, including upload of information by different people in various locations, will perform consistently well and importantly, securely.<br>It is noted that members of the HD community assisted in this careful design process. However, the understanding of this reviewer is that the group who first trialed the app comprised 29 participants of whom most were highly educated.<br>'Half of these individuals were university educated at the undergraduate (6/29, 21%) or postgraduate (9/29, 31%) level, including 2 (N=29, 7%) who had a doctoral degree. Of the remaining participants, 6 (N=29, 21%) had an A-level or bachelor of technology, 4 (N=29, 14%) had a general certificate of secondary education, and 4 (N=29, 14%) had other educational or vocational qualifications. Most participants reported being at the premanifest (8/29, 28%) or early disease stage (17/29, 59%), whereas 4 (N=29, 14%) reported middle or later stages.'<br>These participants are unlikely to reflect the situations of those from varying educational and socioeconomic groups, and for a future larger, more inclusive study or the Clinic population with varying skills. Therefore, as numbers are low in this feasibility study, it would be better to limit clinical inclusion criteria, HD stage

	and CAG expansion lengths (or a Cap score) and aim to include a more varied educational and socioeconomic group.<br>That the MRI scan sequences could provide potentially valuable information supporting the intervention is not in doubt, but the 'ample evidence' quoted relies on very small numbers. The studies referenced in the rebuttal included, after exclusions, 21 HD (4 could not undertake cognitive testing) and 11 controls. In a separate publication, 8 with HD and 9 controls completed these studies and in another (ref 20) there were 5 participants.<br>It will be an important finding if the researchers demonstrate changes on MRI after 10 to 15 minutes of daily drumming for 8 weeks. It is unclear whether the MRI component sequences require a reporter's interpretation and if so whether the reporter (if required) will be blind to the HD DRUM intervention status of the participant.<br>Research in HD is complex. If clinical assessments are unblinded, a significant chance of bias remains on the part of the examiner, and the participants. Separating participants by one TFC point remains an issue. The inclusion of components of HD ISS within Enroll-HD is noted.<br>Usual activity could be a confounder but also of interest and needs documentation. For example, is playing a computer game or the piano 15 minutes a day just as valuable for people with those skills?<br>The study requires collecting and uploading personal information and the participants undergo a procedure with some risks. An independent witness' signature further ensures

**REVIEWER**	Kulisevsky, Jaime<br>Movement Disorders Unit, Neurology Department, Hospital de la Santa Creu i Sant Pau, Universitat Autònoma de Barcelona
**REVIEW RETURNED**	02-Apr-2024

**GENERAL COMMENTS**	The authors have adequately addressed the questions raised by this reviewer. In my opinion, the manuscript has improved and can be accepted.

VERSION 2 – AUTHOR RESPONSE

<u>Responses Reviewer 1's remaining queries:</u>

Ad 1. The authors address some points raised by this reviewer. However, a number remain unresolved in this reviewer's opinion, despite the rebuttal. In summary, unblinded assessments and wide inclusion criteria and reliance on TFC.
As well as all the other stated aims, reliability for this purpose means whether this app, including upload of information by different people in various locations, will perform consistently well and importantly, securely.

We thank the reviewer for taking the time to reassess our rebuttal. We would like to emphasise, that this is a manuscript detailing the protocol for a feasibility trial. The trial is open to recruitment, and we have now recruited around 80% of the target sample, so major protocol changes at this point will have little additional scientific benefit.

As previously stated, within this feasibility trial, many design aspects including the reliability of the app are subject to exploratory investigation to see what would be required, in terms of trial design, for an efficient and inclusive effectiveness trial.

With regards to the consistency and security of the data upload, coded (with a sixteen-digit-letter-string tablet ID) data that do not contain any personal or identifiable information are uploaded automatically to a password protected project-specific space on Google Firebase (Google LLC) when the tablet is connected to the internet using hypertext transfer protocol secure (HTTPS) data encryption at rest and during transit (please see pages 12 and 19 in the manuscript). We have found this procedure reliable across devices and users.

Ad 2. It is noted that members of the HD community assisted in this careful design process. However, the understanding of this reviewer is that the group who first trialed the app comprised 29 participants of whom most were highly educated.

'Half of these individuals were university educated at the undergraduate (6/29, 21%) or postgraduate (9/29, 31%) level, including 2 (N=29, 7%) who had a doctoral degree. Of the remaining participants, 6 (N=29, 21%) had an A-level or bachelor of technology, 4 (N=29, 14%) had a general certificate of secondary education, and 4 (N=29, 14%) had other educational or vocational qualifications. Most participants reported being at the premanifest (8/29, 28%) or early disease stage (17/29, 59%), whereas 4 (N=29, 14%) reported middle or later stages.' These participants are unlikely to reflect the situations of those from varying educational and socioeconomic groups, and for a future larger, more inclusive study or the Clinic population with varying skills. Therefore, as numbers are low in this feasibility study, it would be better to limit clinical inclusion criteria, HD stage and CAG expansion lengths (or a Cap score) and aim to include a more varied educational and socioeconomic group.

We agree with the reviewer that the initial test sample was limited in terms of broad demographics of the population, which is currently largely true of most clinical research.

We would like to clarify though that the demographic information quoted here by the reviewer relates to

29 participants who completed a survey about the use of digital technologies in people with HD, that was

collected prior to the development of the training application, i.e., these individuals did not trial the app (please see Metzler-Baddeley et al JMIR 2023, doi:10.2196/48395). The app was trialed by 12

individuals in the Cardiff University HD clinic, 8 or which were people with HD at premanifest, early manifest, and moderate manifest stages, 2 carers and 2 clinical staff members.

Any future effectiveness trial would be designed in line with National Institute of Health Research (NIHR) INCLUDE (NIHR-INCLUDE https://sites.google.com/nihr.ac.uk/include/home/guidance) guidance to ensure broad inclusivity across ethnicity, religion, education and socio-economic status. The reviewer has suggested that narrowing the clinical inclusion criteria would enable recruitment of a more varied educational and socio-economic cohort. In this instance we disagree. As the study is being conducted at a single site, with two additional active Patient Identification Centre, our potential recruitment population is already relatively limited, and we would start to exclude other demographics. Further, narrowing the clinical inclusion criteria would likely bring this into line with the inclusion/exclusion criteria of other Investigational Medicinal Products (IMP) trials active at these sites- thus further diluting the available sample for recruitment. As we have now recruited ~80% of the sample, we believe that changing the inclusion/ exclusion criteria at this point would have little impact on the demographics of the recruited sample. However, we will record this demographic information and report as part of the cohort baseline characteristics. This will inform recruitment strategies and inclusion criteria for any future effectiveness trial.

Ad 3. That the MRI scan sequences could provide potentially valuable information supporting the intervention is not in doubt, but the 'ample evidence' quoted relies on very small numbers. The studies referenced in the rebuttal included, after exclusions, 21 HD (4 could not undertake cognitive testing) and 11 controls. In a separate publication, 8 with HD and 9 controls completed these studies and in another (ref 20) there were 5 participants.
It will be an important finding if the researchers demonstrate changes on MRI after 10 to 15 minutes of daily drumming for 8 weeks. It is unclear whether the MRI component sequences require a reporter's interpretation and if so whether the reporter (if required) will be blind to the HD DRUM intervention status of the participant.

As mentioned above we have already collected MRI data of ~80% of the sample. We have found MRI data acquisition well-tolerated. Only one participant allocated to the control group dropped out of the study and did not attend the second MRI assessment. Initial analysis of the baseline MRI data has revealed novel information about microstructural tissue changes in the striatum in the living HD brain with the potential of providing novel imaging markers to track disease progression and provide imaging outcome measures in IMP trials.

We are acquiring quantitative microstructural MRI data on an ultra-strong gradient (300mT/m) 3T system. Validated automatic pipelines are being used to preprocess the data and fit biophysical models that cannot be influenced by the researcher running the analyses. The MRI data are interpreted under supervision of a

team of world-leading experts in these advanced techniques with more than 20 years of experience in MRI methods development and clinical applications and blind to the HD DRUM intervention status of

the participants (Professor Derek Jones, Director of CUBRIC, Professor Mara Cercignagni, Head of MRI at CUBRIC, Dr Marco Palombo, Associate Professors and Senior Research Fellow).

Ad 4. Research in HD is complex. If clinical assessments are unblinded, a significant chance of bias remains on the part of the examiner, and the participants. Separating participants by one TFC point remains an issue. The inclusion of components of HD ISS within Enroll-HD is noted.

We acknowledge and agree with the reviewer's comment around bias of unblinded assessments. We agree that unblinding due to the nature of the intervention will affect the validity of the study and this is mentioned in the Strengths and Limitation and blinding sections. However, we attempt to limit any confounding effects of unblinding by randomizing participants into groups only after completion of all baseline assessments, by employing objective computerized assessments of cognitive and motor functions, that cannot be influenced by the researcher and by independent analyses of the Q-motor assessments blinded to groups. Further assessments such as the PHQ-8 and acceptability questionnaire are participant completed and not subject to assessor rating, so the potential for bias to be introduced by the assessor is limited. Similar to our response to the previous point, now that the majority of assessments have been performed, adding in blinded assessment at this point is not likely to confer any scientific benefit. The manuscript already details the justification for non-blinding and noted this as a limitation in a way that satisfies SPIRIT guidance.

Ad 5. Usual activity could be a confounder but also of interest and needs documentation. For example, is playing a computer game or the piano 15 minutes a day just as valuable for people with those skills?

We agree that usual activity could be a confounder but also of interest and will take the need for documentation into consideration when planning a future larger RCT into the clinical effectiveness of the training app.

Ad 6. The study requires collecting and uploading personal information and the participants undergo a procedure with some risks. An independent witness' signature further ensures [we were unsure if this sentence was complete?]

Participant usage of the app is recorded within the software and uploaded automatically, however, as explained under point 1 this information is not characterised as identifiable or personal data within the GDPR. Collection of health-related personal data is completed at on-site assessments and there are no requirements for participants to upload personal data. A full risk assessment of the trial has been carried out, including a data impact protection assessment and reviewed and the study has been characterised as low-risk.

We respectfully disagree with the requirement for an additional signature as only people with capacity to consent are included in this study and our ethical approval does not require a witness to the participant's signature other than the researcher. Requirement for an additional signature could potentially exclude those visiting clinic on their own and it is not within our local practice to insist on independent sign off on consent on participants who have been deemed to have capacity, particularly for low-risk non-pharmacological interventions.
